# Highly Selective Fluorescent Sensing of Proteins Based on a Fluorescent Molecularly Imprinted Nanosensor

**DOI:** 10.3390/s131012994

**Published:** 2013-09-26

**Authors:** Qiliang Deng, Jianhua Wu, Xiaorui Zhai, Guozhen Fang, Shuo Wang

**Affiliations:** 1 Department of Science, Tianjin University of Science and Technology, Tianjin 300457, China; E-Mail: yhqld@tust.edu.cn; 2 Key Laboratory of Food Nutrition and Safety, Ministry of Education of China, Tianjin Key Laboratory of Food Nutrition and Safety, Tianjin University of Science and Technology, Tianjin 300457, China; E-Mails: Wu5287211@163.com (J.W.); xiaoruizhaizhai@163.com (X.Z.); tjfanggz@sina.com (G.F.)

**Keywords:** molecularly imprinted polymers, protein, fluorescent sensing

## Abstract

A fluorescent molecularly imprinted nanosensor was obtained by grafting imprinted polymer onto the surface of multi-wall carbon nanotubes and post-imprinting treatment with fluorescein isothiocyanate (FITC). The fluorescence of lysozyme-imprinted polymer (Lys-MIP) was quenched more strongly by Lys than that of nonimprinted polymer (NIP), which indicated that the Lys-MIP could recognize Lys. The resulted imprinted material has the ability to selectively sense a target protein, and an imprinting factor of 3.34 was achieved. The Lys-MIP also showed selective detection for Lys among other proteins such as cytochrome C (Cyt C), hemoglobin (HB) and bovine serum albumin (BSA) due to the imprinted sites in the Lys-MIP. This approach combines the high selectivity of surface molecular imprinting technology and fluorescence, and converts binding events into detectable signals by monitoring fluorescence spectra. Therefore, it will have further applications for Lys sensing.

## 1. Introduction

Artificial synthetic materials capable of protein recognition are very important for the life sciences, especially for the study of proteomics and clinical diagnostics. Although biomolecules such as antibodies, receptors, enzymes, and various aptamers have shown great potential for these purposes, the limites availability of sources has hampered their wide application. Recently, more attention has been paid to artificial materials mimicking biomolecules. Among those, molecularly imprinted polymers (MIPs) synthesized by molecular imprinting are one of the most popular materials due to their simplicity, low-cost, high selectivity and high affinity for target molecules [1–3]. Molecular imprinting is considered as an elegant template polymerization technique to prepare synthetic materials with pre-determined recognition sites for target molecules. MIPs for various small molecules have been successfully obtained by co-polymerization of functional monomer and cross-linker around the template molecules [4–8]. Imprinting of proteins is attracting more and more attention, albeit it remains a challenging task [9–11].

Selective fluorescent sensing of target molecules by combining MIP with a fluorescent reagent or quantum dots have been proven to be highly desirable due to the high selectivity of MIP and sensitivity of fluorescence [12–15]. MIPs with fluorescent reporters were usually prepared by coating quantum dots with the MIP or co-polymerizing a fluorescent monomer in the molecular imprinting process. Thus, many fluorescent monomers or quantum dots are positioned outside the imprinting cavity or encapsulated into the materials, and this leads to high background fluorescence. In order to alleviate this shortcoming, a post-imprinting treatment strategy, in which MIPs was modified after the imprinting process with a minimum amount of a fluorescent molecule, has been developed [16,17]. However, a specifically designed monomer is a prerequisite for the development of such sensors, and this has also hindered further applications of this approach.

Multi wall carbon nanotubes (MWCNTs) are considered ideal supporting materials due to their extremely large surface area and unique mechanical properties [18,19]. Recently, MIPs have been coated on the surface of functionalized MWCNTs. Usually, MWCNTs were first functionalized with initiator or vinyl groups, and then MIPs were immobilized on their surface [20,21]. Another strategy is immobilization of MIPs on the surface of carboxylic acid-functionalized MWCNTs [22]. The resulting MIPs□CNTs showed high extraction efficiency, short binding times and the easy accessibility of the template molecule.

In this communication, we developed a fluorescent imprinted nanosensor for protein detection by grafting an imprinted polymer onto the surface of MWCNTs, and a subsequent post-imprinting treatment. The binding behavior of the resulted material was investigated by the changes of its fluorescence signal. Practical applications of the fluorescent polymer were also determined.

## 2. Experimental Section

MWCNTs (OD 20–30 nm, –COOH content 1.23 wt%) were obtained from Chengdu Organic Chemicals Co. Ltd., Chinese Academy of Sciences (ChengDu, China). Lysozyme (Lys, molecular weight 14.4 kDa, pI 10.8), Cytochrome C (Cyt C, molecular weight 12.2 kDa, pI 10.2–10.8), myoglobin (Mb, molecular weight 17.5 kDa, pI 6.8–7.2), hemoglobin (Hb, molecular weight 64.5 kDa, pI 6.8–7.0) and bovine serum albumin (BSA, molecular weight 67.0 kDa, pI 4.7) were purchased from Sigma Biochemical Co. Ltd. (Shanghai, China). Fluorescein isothiocyanate isomer 1 (FITC, C_21_H_11_NO_5_S) was from Sigma Aldrich Co. Ltd. (Shanghai, China). Acrylamide (AAm) was the product of Sangon Biotech Co. Ltd. (Shanghai, China). N,N'-methylenebisacrylamide (MBA), ammonium persulfate (APS) and acetic acid were purchased from Concord Co. Ltd. (Tianjin, China). Sodium dodecyl sulfate (SDS) was obtained from BiO basic Inc. (Shanghai, China). N,N,N',N'-tetramethylethylenediamine (TEMED) was the product of Sinopharm Chemical Reagent Co. Ltd. (Shanghai, China). Doubly deionized water (DDW, 18.2 MΩ·cm^−1^) was prepared from a Walter Pro water system (Labconco, Kansas City, MO, USA). Egg white samples were obtained from hen egg white. All other reagents used in this study were of analytical grade.

Fluorescence spectra were measured by a F-4500 fluorescence spectrophotometer from Hitachi Co. Ltd. (Hitachi, Japan). A Fourier transform infrared spectrometer (FT-IR) (4,000–400 cm^−1^) (Vector 22, Bruker, Bremen, Germany) was used for the characterization of the synthesized polymers.

MIP grafted to carbon nanotube was prepared as follows: MWNTs (0.1000 g) and phosphate buffer solution (PBS, 3.0 mL, 0.01 mol·L^−1^, pH = 6.8) were mixed and ultrasonicated for 1 h to form the first aqueous phase. Lys (0.0220 g) and AAm (0.8800 g) were dissolved in PBS (3.0 mL) to form the second aqueous phase. The first and second aqueous phases were magnetically stirred for 3 h in a glass flask. Then MBA (0.1200 g) and PBS (2.0 mL) were added into the glass flask. After 20 min, APS (0.0300 g) was added into the glass flask to magnetically stir for 10 min. The mixture was purged with nitrogen for 15 min to displace dissolved oxygen. Finally, TEMED (100 μL, 20 v/v%) was added into the mixture which was incubated for 24 h at 25 °C. Then, the polymer was washed with solution of SDS/HAc (10 w/v%, 10 v/v%) to remove Lys, followed by extensive washing with doubly deionized water until no free protein was detectable using a UV-visible spectrophotometer at a wavelength of 281.0 nm. Nonimprinted polymer (NIP) was also prepared for comparison using the same procedure but without Lys. Finally, the polymers were dried by freeze drying for 24 h.

Lys synthesized materials (0.1000 g) were added into FITC (10.0 mL, 500 mM) to magnetically stir at room temperature for 24 h in a glass flask. Then, the mixture was washed with doubly deionized water to remove unreacted FITC until no fluorescence spectrum was detected using fluorescence spectrophotometer at the excitation wavelength of 493.0 nm. Finally, the modified materials were dried by freeze drying for 24 h.

All the fluorescence measurements were carried out with the excitation wavelength of 493.0 nm and the emission wavelength of 518.0 nm. The slit widths of excitation and emission were 2.5 and 5.0 nm, respectively.

The equilibrium adsorption experiments were investigated with the MIP and NIP, respectively. Lys solution was prepared with PBS (0.01 mol·L^−1^, pH = 6.8). In each adsorption test, the material (0.0050 g) and Lys solution (0.5 mL) were added into 1.5 mL Eppendorf tubes which were shaken in a dark place at room temperature for 24 h. Then, the mixture was diluted 200 times. After shaking equably, the mixture was determined quickly by fluorescence spectrophotometer.

For dynamic adsorption experiments, the material (0.0050 g) and Lys solution (0.5 mL, 200 mg·L^−1^) were agitated for different times (15, 30, 60, 120, 180, 300, 420 and 600 min) in a dark place at room temperature.

The selectivity of the MIP for Lys and the competitive proteins (Cyt C, Hb and BSA) was evaluated. The MIP or NIP was mixed with protein solution (0.5 mL) containing Cyt C:Lys, Hb:Lys or BSA:Lys at different ratios (0:2, 1:2, 2:2, 3:2, 4:2, 5:2, 6:2). After adsorption was completed, the mixture was diluted 200 times. The mixed system was detected by the fluorescence spectrophotometer.

Chicken egg white was diluted 15-fold with PBS solution containing 0.25 mol·L^−1^ NaCl. The diluted solution was ultrasonicated for 5 min and centrifuged at 4,000 rpm for 10 min. The supernatant solution was diluted 1.25, 1.70, 2.50, and 5.00 times in sequence as a Lys source. MIP or NIP (0.0050 g) was added to different concentrations of Lys source, and shaken in a dark place for 5 h. The mixture was diluted 200 times and determined by the fluorescence spectrophotometer.

## 3. Results and Discussion

### 3.1. Preparation and Characterization of the Lys-MIP

**Scheme 1.**
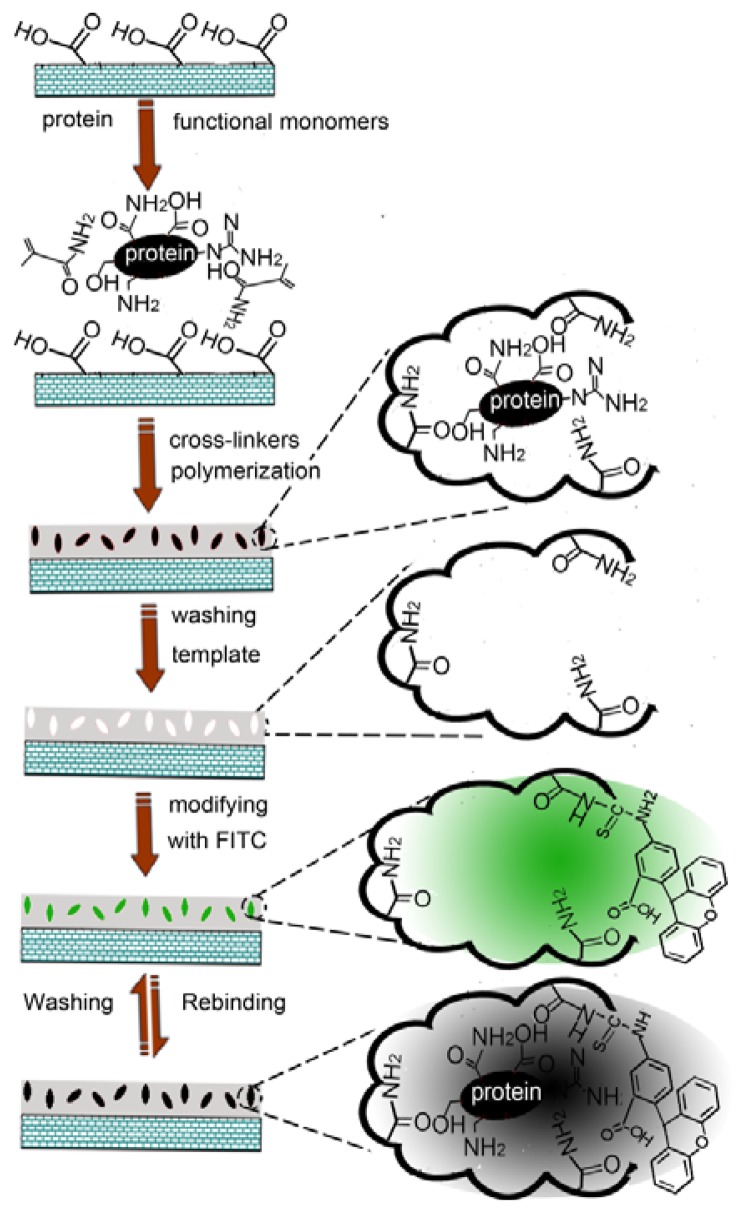
A diagram to illustrate the preparation of the fluorescent imprinted nanotubes and the protein detection mechanism.

In this communication, we have developed a fluorescent imprinted nanosensor for protein detection by grafting an imprinted polymer onto the surface of multi-wall carbon nanotubes, and a subsequent post-imprinting treatment, in which no special functional monomer is involved. Scheme 1 presents a schematic diagram to illustrate the preparation of the fluorescent imprinted sensor and the protein detection mechanism.

Multi-wall carbon nanotubes, which have been widely applied in many fields due to their unique mechanical properties and extremely large surface area, were chosen as matrix. A commercially available monomer, AAm, was chosen to synthesize imprinted polymers for Lys via chemical oxidation polymerization by persulfate ammonium. The polymer was characterized by SEM and FTIR (Figures S1 and S2, Supplementary Material). The general concept used in this approach lies in the fact that fluorescent element is introduced into the imprinting cavity by the reaction of the amino group of the functional monomer with the isothiocyano group of FITC, and subsequently the template protein binding leads to fluorescence quenching. This quenching may be attributed to the interaction of the template protein with the FITC in the imprinting cavity.

**Figure 1.**
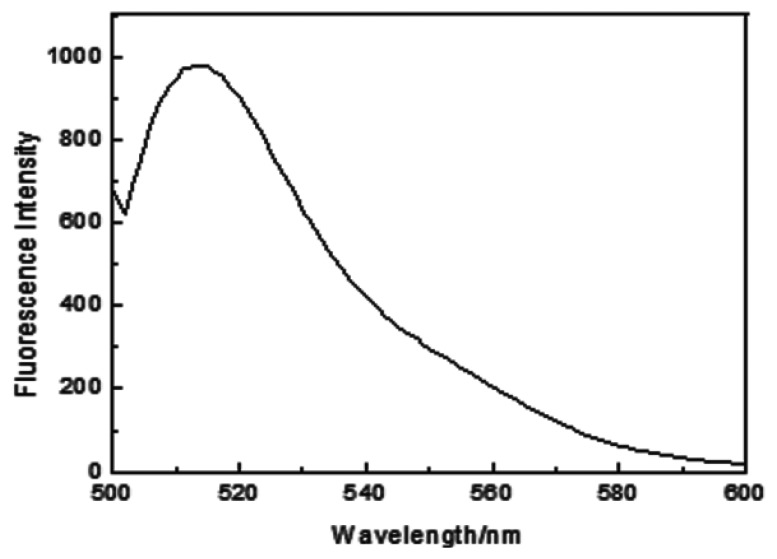
Fluorescence emission spectrum (λex = 493.0 nm) of MIP modified with FITC in PBS (0.01 mol·L^−1^, pH = 6.8) solution.

**Figure 2.**
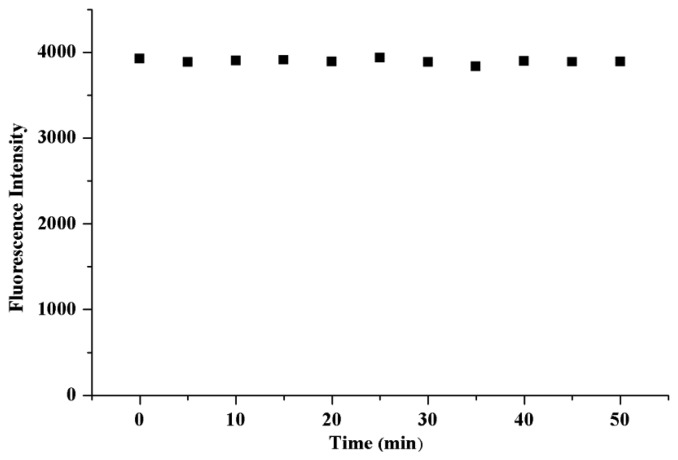
Fluorescence stability of the MIP in PBS solution.

MIP modified with FITC showed an emission maximum at 518.0 nm, which was attributed to fluorescein (Figure 1). A similar fluorescent emission was also observed for NIP (data not shown). The fluorescence stability of the modified material in PBS was determined (Figure 2). The result indicated that the fluorescence of the modified material was stable and the fluorescence intensity wasn't changed within 50 min, which ensured reliability of the protein determination.

### 3.2. Affinity of the Lys-MIP

**Figure 3.**
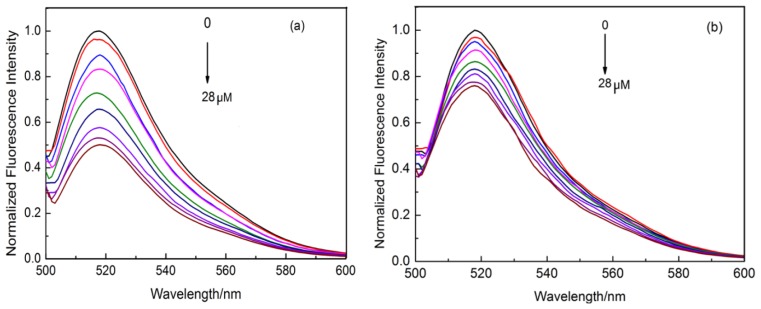
Fluorescence spectra (λex = 493.0 nm) of the MIP (**a**) and NIP (**b**) with increasing Lys concentration in the PBS solution.

In order to prove the recognition ability of the Lys-MIP versus that of the NIP, the recognition of Lys was performed in PBS solution with different concentrations ranging from 0 to 28 μM (Figure 3). It could be seen that the fluorescence intensity of MIP was quenched gradually with the increasing concentration of Lys (Figure 3a), which indicated that Lys has been adsorbed onto the imprinted cavities conjugated with the FITC in the Lys-MIP. However, the change of fluorescence intensity of the NIP was less sensitive than that of the NIP by Lys at the same concentration (Figure 3b).

**Figure 4.**
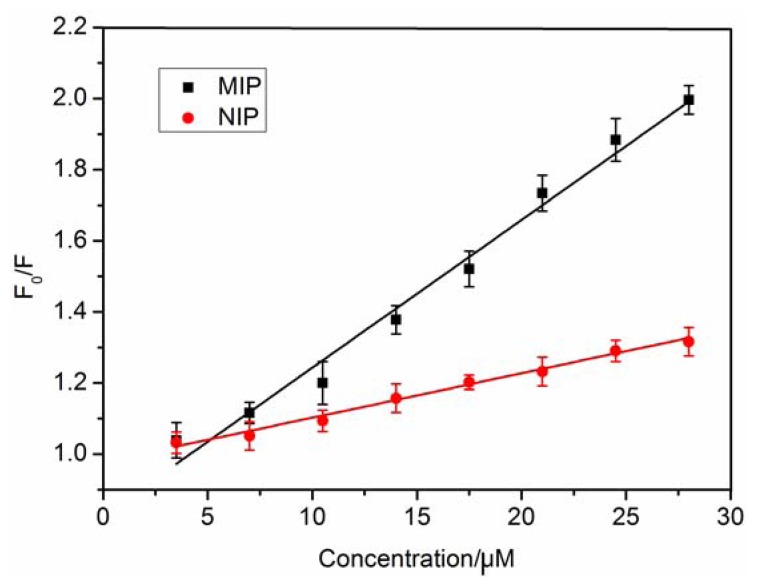
Stern-plot of MIP and NIP.

The data was further fitted with the Stern-Volmer equation: (F_0_/F) = 1 + K_SV_ [A], where F_0_ and F are the fluorescence intensities in the absence and presence of target molecule, respectively. [A] represents the concentration of the concentration of the target molecule, and K_SV_ is the quenching constant of the target molecule. The result showed that the Stern-Volmer plots of the MIP and NIP exhibited linear relationship when the concentration of Lys was changed from 0 to 28.0 μM (Figure 4). The values of K_SV_ were 0.04158 μM^−1^ and 0.01246 μM^−1^ for MIP and NIP, respectively. The imprinting factor (IF) that is the ratio of the quenching constants (K_SV_-MIP/K_SV_-NIP) is used to evaluate the selectivity of the polymer. The result showed that the IF was 3.34, which is superior to the reported results [14]. In a surface-imprinted polymer, the imprinted sites are on the surface so they provide excellent site accessibility and low mass-transfer resistance for Lys. All these results indicated that the imprinted cavities could greatly improve the fluorescence quenching efficiency by Lys adsorption and enhance the fluorescence spectral responses of FITC to Lys.

**Figure 5.**
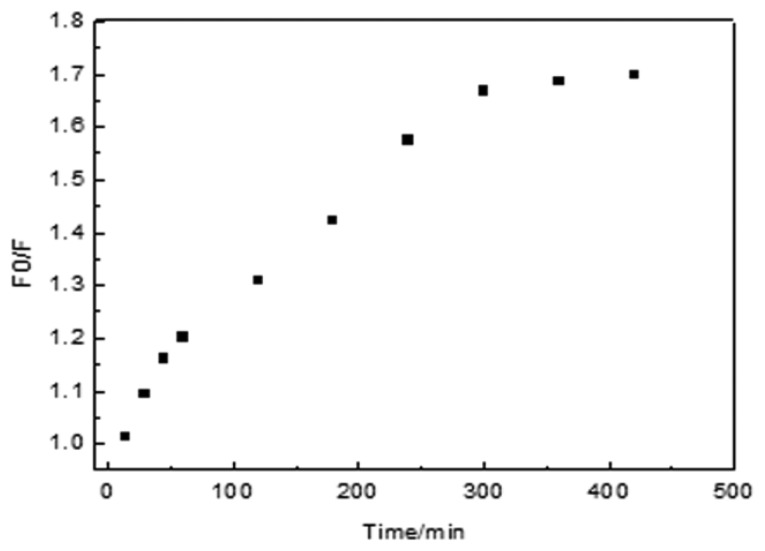
Recognition kinetics of Lys on the MIP.

The rebinding kinetics of MIP for Lys were evaluated and it was found that 70.7% binding was completed within 60 min (Figure 5). The adsorption equilibrium was achieved within 300 min. Then little change was observed after 300 min, which indicated that adsorption process nearly reached equilibrium. This result is probably due to the abundance of imprinted sites situated on the surface of the MIP, so Lys can easily enter the specific binding cavities. After the binding cavities on the surface of the Lys-MIFP are occupied, the penetration of Lys into the polymer becomes much more difficult.

### 3.3. Specificity Experiments

**Figure 6.**
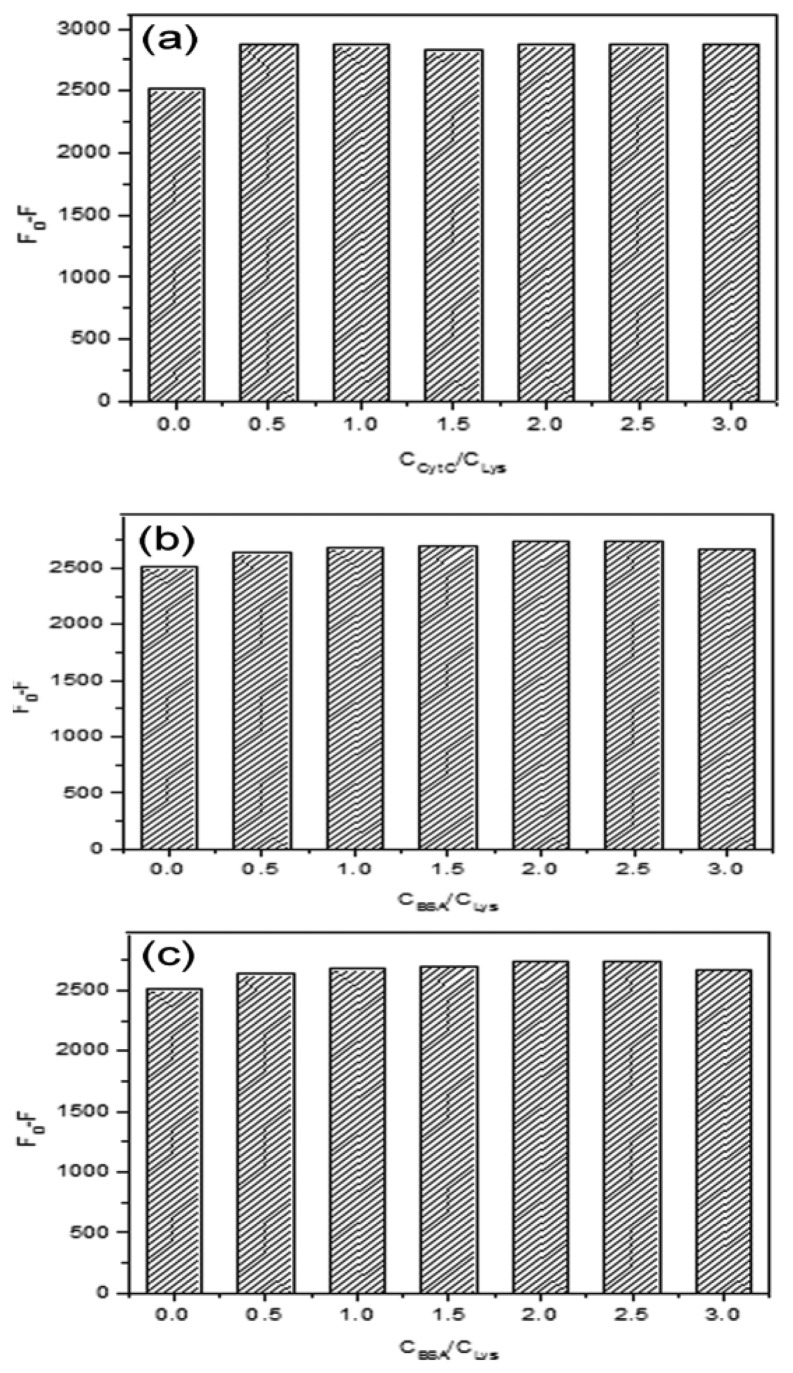
Influence of the competitive proteins Cyt (C_Cyt C_/C_Lys_ 0, 0.5, 1.0, 1.5, 2.0, 2.5, 3.0) (**a**); Hb (C_Cyt C_/C_Lys_ 0, 0.5, 1.0, 1.5, 2.0, 2.5, 3.0) (**b**); and BSA (C_Cyt C_/C_Lys_ 0, 0.5, 1.0, 1.5, 2.0, 2.5, 3.0) (**c**) for the adsorption of Lys (25 μM) on the Lys-MIP.

The response specificity of MIP was further investigated by using Cyt C, Hb and BSA as competitive proteins. These proteins have different molecular weights and isoelectric points. The specificity experiments were carried out by fixing the concentration of Lys (25 μM) and increasing the concentration of Cyt C, Hb or BSA. It can be seen from Figure 6 that there was little change by increasing of the ratio of C_Cyt C_/C_Lys_, C_Hb_/C_Lys_, or C_BSA_/C_Lys_ from 0 to 3.0. The results showed that the sites formed in the MIP matched with the size of Lys, so Lys was bound to the MIP and caused the significant change of fluorescence intensity. As the competitive protein, Cyt C was similar to Lys in molecular weight, but the spatial arrangement was different from Lys, so there was less chance to quench the fluorescence of the MIP. For Hb and BSA, the molecular volume was larger than that of Lys, so access to the imprinted sites was limited by the steric hindrance of the polymer network. Therefore, Hb or BSA couldn't interact with the imprinted cavities specifically.

### 3.4. Detection of Lys in Chicken Egg White by Fluorescence Sensor

**Figure 7.**
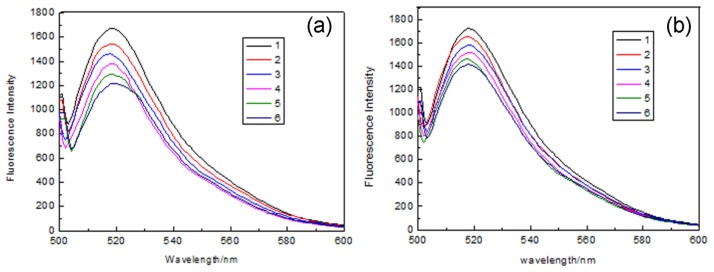
Fluorescence spectra of MIP (**a**) and NIP (**b**) with increasing Lys concentration in egg white. (1) Without addition of egg white solution; (2) dilute egg white solution (1:75); (3) dilute egg white solution (1:37.5); (4) dilute egg white solution (1:25.5); (5) dilute egg white solution (1:18.8); (6) dilute egg white solution (1:15).

**Figure 8.**
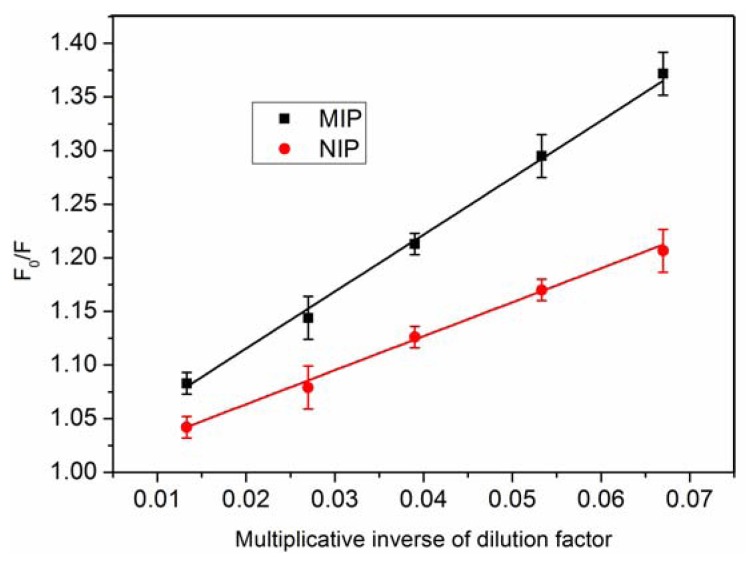
The relationship between F_0_/F and multiplicative inverse of dilution factor.

To explore the use of the fluorescent MIP as the fluorescence sensor, chicken egg white, which contains the template protein, was chosen as a matrix. The fluorescence spectra of MIP and NIP were recorded with the increase of chicken egg white solution concentration. As seen in Figure 7, the fluorescence intensity of the MIP and NIP decreased gradually with increasing egg white concentration. The fluorescence quenching of the MIP was much larger than that of NIP. In order to check the relationship between the fluorescence quenching and the concentration of lysozyme in sample, plots of F_0_/F *versus* multiplicative inverse of dilution factor was shown in Figure 8. The value of F_0_/F is increased with the increase of the value of multiplicative inverse of dilution factors. This also indicated that the fluorescence was significantly quenched with the increase of chicken egg white concentration. The result was consistent with the affinity experiments. Therefore, the fluorescent MIP was expected to be a good material for selective sensing of Lys.

## 4. Conclusions

In summary, a fluorescent MIP nanosensor for selective sensing of Lys was synthesized by a facile post-imprinting treatment, and no specially designed functional monomer was involved. The recognition behaviour of the fluorescent MIP for Lys was evaluated, and the results showed that the recognition effect of the fluorescent MIP was better than that of the fluorescent NIP. The imprinting factor was 3.34. The binding capacity became 70.7% within 60 min, and the adsorption equilibrium was achieved when the adsorption time was 300 min. Specific experiments further showed that the recognition ability of the fluorescent MIP was steady. Sensing experiments with Lys from egg white indicated that the fluorescent MIP could be expected to be a good material for selective sensing of Lys.

## References

[1] Wulff G., Sharhan A. (1972). Über die Anwendung von enzymanalog gebauten Polymeren zur Racemattrennung. Angew. Chem..

[2] Vlatakis G., Anderson L.I., Muller R., Mosbach K. (1993). Drug assay using antibody mimics made by molecular imprinting. Nature.

[3] Hoshino Y., Kodama T., Okahata Y., Shea K.J. (2008). Peptide imprinted polymer nanoparticles: A plastic antibody. J. Am. Chem. Soc..

[4] Flavin K., Resmini M. (2009). Imprinted nanomaterials: A new class of synthetic receptors. Anal. Bioanal. Chem..

[5] Huang Y.P., Liu Z.S., Zheng C., Gao R.Y. (2009). Recent developments of molecularly imprinted polymer in CEC. Electrophoresis.

[6] Lee W.C., Cheng C.H., Pan H.H., Chung T.H., Hwang C.C. (2008). Chromatographic characterization of molecularly imprinted polymers. Anal. Bioanal. Chem..

[7] Maier N.-M., Lindner W. (2007). Chiral recognition applications of molecularly imprinted polymers: A critical review. Anal. Bioanal. Chem..

[8] Jung B.M., Kim M.S., Kim W.J., Chang J.Y. (2010). Molecularly imprinted mesoporous silica particles showing a rapid kinetic binding. Chem. Commun..

[9] Ellen V., Joris P.-S., Martin V.W., Marie-Astrid D., Wim E.H., Cornelus V.N. (2011). Challenges for the effective molecular imprinting of proteins. Biomaterials.

[10] Yi G., Anthony T.P.F. (2008). Too large to fit? Recent developments in macromolecular imprinting. Trends Biotech..

[11] Liu J.X., Yang K.G., Deng Q.L., Li Q.R., Zhang L.H., Liang Z., Zhang Y.K. (2011). Preparation of a new type of affinity materials combining metal coordination with molecular imprinting. Chem. Commun..

[12] Takeuchi T., Mukawa T., Shinmori H. (2005). Signaling molecularly imprinted polymers: Molecular recognition-based sensing materials. Chem. Rec..

[13] Kubo H., Yoshioka N., Takeuchi T. (2005). Fluorescent imprinted polymers prepared with 2-acrylamidoquinoline as a signaling monomer. Org. Lett..

[14] Zhang W., He X.-W., Chen Y., Li W.Y., Zhang Y.K. (2011). Composite of CdTe quantum dots and molecularly imprinted polymer as a sensing material for cytochrome C. Biosens. Bioelectron..

[15] Li H.B., Li Y.L., Cheng J. (2010). Molecularly imprinted silica nanospheres embedded CdSe quantum dots for highly selective and sensitive optosensing of pyrethroids. Chem. Mater..

[16] Tao Z., Tehan E.C., Bukowski R.M., Tang Y., Shughart E.-L., Holthoff W.G., Cartwright A.N., Titus A.-H., Bright F.-V. (2006). Templated xerogels as platforms for biomolecule-less biomolecule sensors. Anal. Chim. Acta.

[17] Sunayama H., Ooya T., Takeuchi T. (2010). Fluorescent protein recognition polymer thin films capable of selective signal transduction of target binding events prepared by molecular imprinting with a post-imprinting treatment. Biosens. Bioelectron..

[18] Huang Q., Gao L. (2003). Immobilization of rutile TiO2 on multiwalled carbon nanotubes. J. Mater. Chem..

[19] An L., Xu W., Rajagopalan S., Wang C., Wang H., Fan Y., Zhang L., Jiang D., Kapat J., Chow L. (2004). Carbon-nanotube-reinforced polymer-derived ceramic composites. Adv. Mater..

[20] Kan X.W., Zhao Y., Geng Z.R., Wang Z.L., Zhu J.-J. (2008). Composites of multiwalled carbon nanotubes and molecularly imprinted polymers for dopamine recognition. J. Phys. Chem. C.

[21] Lee H.Y., Kim B.S. (2009). Grafting of molecularly imprinted polymers on iniferter-modified carbon nanotube. Biosens. Bioelectron..

[22] Zhang M.S., Huang J.R., Yu P., Chen X. (2010). Preparation and characteristics of protein molecularly imprinted membrance on the surface of multiwalled carbon nanotubes. Talanta.

